# Safety and Efficacy of Scanning Ultrasound Treatment of Aged APP23 Mice

**DOI:** 10.3389/fnins.2018.00055

**Published:** 2018-02-07

**Authors:** Gerhard Leinenga, Jürgen Götz

**Affiliations:** Clem Jones Centre for Ageing Dementia Research, Queensland Brain Institute, University of Queensland, Brisbane, QLD, Australia

**Keywords:** Alzheimer's disease, microbleeds, scanning ultrasound, amyloid-β, microglia, therapy

## Abstract

Deposition of amyloid-β (Aβ) peptide leads to amyloid plaques that together with tau deposits characterize the brains of patients with Alzheimer's disease (AD). In modeling this pathology, transgenic animals such as the APP23 strain, that expresses a mutant form of the amyloid precursor protein found in familial cases of AD, have been instrumental. In previous studies, we have shown that repeated treatments with ultrasound in a scanning mode (termed scanning ultrasound or SUS) were effective in removing Aβ and restoring memory functions, without the need for a therapeutic agent such as an Aβ antibody. Considering that age is the most important risk factor for AD, we extended this study in which the mice were only 12 months old at the time of treatment by assessing a cohort of 2 year-old mice. Interestingly, at this age, APP23 mice are characterized by cerebral amyloid angiopathy (CAA) and the presence of occasional microbleeds. We found that SUS in aged mice that have been exposed to four SUS sessions that were spread out over 8 weeks and analyzed 4 weeks later did not show evidence of increased CAA or microbleeds. Furthermore, amyloid was reduced as assessed by methoxy-XO4 fluorescence. In addition, plaque-associated microglia were more numerous in SUS treated mice. Together this adds to the notion that SUS may be a treatment modality for human neurodegenerative diseases.

## Introduction

Life expectancy has increased dramatically in the past decades, owing to general lifestyle improvements and better medication; however, the ensuing dramatic demographic shift in age distribution is associated with an increased prevalence of diseases such as cerebrovascular disease, cancer and dementia (Leinenga et al., 2017). In contrast to cerebrovascular disease and cancer, where non-pharmacological therapies play important roles, the focus in neurological disease research has been on drug discovery, despite the fact that delivery of drugs to the brain presents a particular challenge due to the presence of the blood-brain barrier (BBB). This barrier not only prevents any exchange between the blood and nervous tissue, thereby protecting the brain parenchyma from circulating factors, and restricting access of pathogens and immune cells to the brain (Wong et al., 2013); it also allows only a small subset of small-molecule drugs to enter the brain via lipid-mediated free diffusion, posing a major problem for drug delivery (Pardridge, 2012).

Whether the BBB in neurodegenerative diseases (different from stroke and glioma) is leaky remains a matter of debate (Leinenga et al., 2016). The notion that the BBB becomes leaky in neurological disease disregards the fact that the brain is not completely sealed off from the periphery when healthy. Active exchange between the blood and the brain under normal physiological conditions is indicated by the fact that, for example, steady-state brain levels of peripherally administered antibodies are approximately 0.1% of those in the plasma (Levites et al., 2006). Such evidence does not mean that the BBB is not compromised under certain pathological conditions, but rather that the precise nature of the damage is incompletely understood (Gilad et al., 2012; Krueger et al., 2013; Knowland et al., 2014). Although cellular senescence can impair BBB function (Murugesan et al., 2012; Lucke-Wold et al., 2014), several lines of evidence from studies of Alzheimer's disease (AD) show that disruption of the BBB in neurodegenerative disease should not be assumed, with a study published in 2015 reporting a lack of widespread BBB disruption in several mouse models of Alzheimer's disease, and a similar occurrence of infarcts (one indication of BBB breakdown) in patients with AD and age-matched healthy controls (Bien-Ly et al., 2015).

In order to manipulate the opening of the BBB for targeted drug or gene delivery, non-thermal ultrasound can be used to capitalize on the interaction between ultrasound and microscopic bubbles of gas (microbubbles) in tissue or fluids (Sirsi and Borden, 2009). Injection of preformed, commercially available microbubbles is used to ensure biological effects even at low acoustic pressures (Konofagou, 2012). These microbubbles are routinely used for contrast-enhanced ultrasound imaging. They are biologically inert and have a gas core encapsulated by a thin shell of lipid or polymer, and a diameter <10 μm. Ultrasound causes them to cavitate, i.e., expand and contract, resulting in vessel wall displacement (McDannold et al., 2006; Caskey et al., 2007; Raymond et al., 2007). Displacement causes transient opening of tight junctions because of the disintegration of the associated junction complexes. This transiently facilitates transport across the BBB (Sheikov et al., 2008).

The two key molecules implicated in AD are Aβ and tau (Li and Götz, 2017b). Aβ is a small peptide derived by proteolytic cleavage from the larger amyloid precursor protein (APP) that aggregates and forms histological lesions known as amyloid plaques. While AD is characterized by the intraparenchymal deposition of amyloid-β in plaques, a majority of AD patients also exhibit deposition of amyloid in blood vessel walls, mainly small arteries, a pathology called cerebral amyloid angiopathy abbreviated CAA (Attems et al., 2005). CAA increases the risk of intracerebral hemorrhage by making amyloid-laden blood vessels more susceptible to damage (Banerjee et al., 2017). Tau is a microtubule-associated protein that forms neurofibrillary tangles. Tau pathology is found not only in AD but also in many diseases collectively termed tauopathies which includes a major subset of FTLD termed FTLD-Tau. Tau is required for Aβ toxicity (Ittner et al., 2010), and Aβ initiates the *de novo* protein synthesis of tau in the somatodendritic domain (Li and Götz, 2017a).

We and others have previously shown that microbubble-mediated BBB opening without any therapeutic agent reduces an Aβ pathology in APP mutant mice (Jordão et al., 2013; Leinenga and Götz, 2015). By weekly treatments of 12 month-old APP23 mice between 5 and 8 times using ultrasound in a scanning mode (SUS for scanning ultrasound), Aβ species ranging from monomers to oligomers to high molecular weight species could be cleared effectively, plaques were partially cleared, and memory functions were restored in three complementary tests (Leinenga and Götz, 2015). As an underlying clearance mechanism we had identified the internalization of Aβ by brain-resident dormant microglial cells that became activated by unidentified blood-borne factors that entered the brain as a consequence of the transient opening of the BBB. Interestingly, we found that tau pathology (being mostly intraneuronal) could also be partially cleared with this approach as shown in the pR5 transgenic mouse model of tauopathy which accumulates hyperphosphorylated tau in neurons (Nisbet et al., 2017). Long-term safety has been demonstrated in wild-type mice (Hatch et al., 2016), and an application to proteinopathies more generally can be envisaged (Leinenga et al., 2017; Li and Götz, 2017b). Here, we aimed to determine whether the cerebral amyloid angiopathy (CAA) and microbleeds that characterize aged APP23 mice (Calhoun et al., 1999) would be features that are augmented by a SUS treatment, and also, whether there would be a reduction in amyloid in mice with advanced amyloid pathology.

## Materials and methods

### Animal models and ethics

APP23 mice express hAPP751 together with the Swedish double mutation under control of the neuron-specific mThy1.2 promoter (Sturchler-Pierrat et al., 1997). APP23 mice are characterized by amyloid plaque formation mainly in the cortex, as well as associated memory deficits, and cerebral amyloid angiopathy as they age (Calhoun et al., 1999; Winkler et al., 2001). They also display a premature lethality that comes to a hold when they reach a few months of age (Ittner et al., 2010). Animal experimentation was approved by the Animal Ethics Committee of the University of Queensland (approval number QBI/412/14/NHMRC).

### SUS equipment

**Figure 1:**
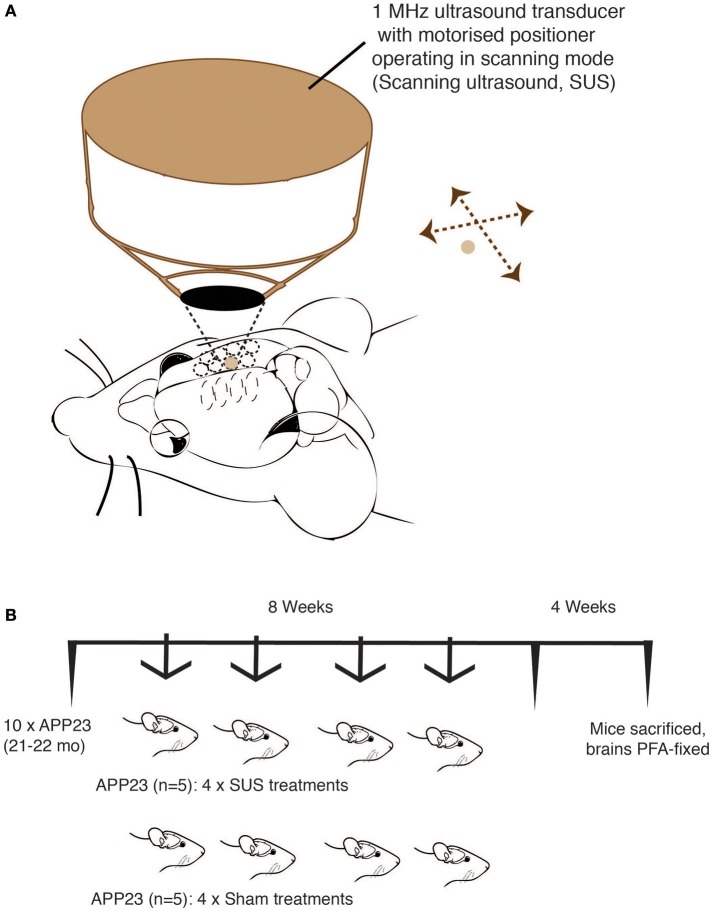
Treatment scheme for of aged APP23 mice. Scanning ultrasound (SUS) approach using the TIPS system (Philips Research) that consists of an annular 1 MHz transducer operating on a motorized stage to allow the sequential treatment of multiple foci until a total coverage of the mouse forebrain is achieved. **(A)** Treatment scheme of 21–22 month-old APP23 mice (mixed gender) divided into two matching groups (*n* = 5 per group). **(B)** One group received 4 SUS treatments spread out over an 8 week period, whereas the other group received 4 sham treatments (anesthetics and microbubbles only) over the same period. Four weeks after the final treatment, the mice were sacrificed and their forebrains fixed in 4% PFA for histology.

An integrated focused ultrasound system was used (Therapy Imaging Probe System, TIPS, Philips Research) (Seip et al., 2010). The system consisted of an annular array transducer with a natural focus of 80 mm, a radius of curvature of 80 mm, a spherical shell of 80 mm with a central opening of 31 mm diameter, a 3D positioning system, and a programmable motorized system to move the ultrasound focus in the x and y planes to cover the entire brain. A coupler mounted to the transducer was filled with degassed water and placed on the head of the mouse with ultrasound gel for coupling, to ensure propagation of the ultrasound to the brain (Figure 1A). The focal zone of the array was an ellipse of approximately 1.5 × 1.5 × 12 mm.

### Production of microbubbles

In-house prepared microbubbles comprising a phospholipid shell and octafluoropropane gas core were used. 1,2-distearoyl-*sn*-glycero-3-phosphocholine (DSPC) and 1,2-distearoyl-sn-glycero-3-phosphoethanolamine-N-[amino(polyethylene glycol)-2000] (DSPE-PEG2000) (Avanti Polar Lipids) were mixed at a 9:1 molar ratio dissolved in chloroform (Sigma) and the chloroform solvent was evaporated under vacuum. The dried phospholipid cake was then dissolved in PBS with 10% glycerol to a concentration of 1 mg lipid/ml and heated to 55°C in a sonicating water bath. The solution was placed in 1.5 ml glass HPLC vials and the air in the vial was replaced with octafluoropropane (Arcadophta). Microbubbles were generated on the day of the experiment by agitation in a dental amalgamator at 4,000 rpm for 40 s. Microbubbles were observed under a microscope to be polydispersed and under 10 μm in size at a concentration of 1–5 × 10^8^ microbubbles/ml.

### SUS application

Mice were anesthetized with ketamine (100 mg/kg) and xylazine (10 mg/kg) and the hair on the head was shaved and depilated. Mice were injected intravenous retro-orbitally with 1 μl/g body weight of microbubble solution and then placed under the ultrasound transducer with the head immobilized. We have previously found that retro-orbital injections give identical results as tail vein injections and are technically easier to perform. Parameters for the ultrasound delivery were 0.7 MPa peak rarefactional pressure, 10 Hz pulse repetition frequency, 10% duty cycle, and a 6 s sonication time per spot. The focus of the transducer had dimensions of 1.5 × 12 mm in the transverse and axial planes, respectively. The motorized positioning system moved the focus of the transducer array in a grid with 1.5 mm spacing between individual sites of sonication so that ultrasound was delivered sequentially to the entire brain as described (Nisbet et al., 2017). Mice typically received a total of 24 spots of sonication in a 6 × 4 raster grid pattern. For sham treatment, mice received all injections and were placed under the ultrasound transducer, but no ultrasound was emitted.

### Tissue processing

Mice were deeply anesthetized with pentobarbitone before being perfused with 30 ml of phosphate-buffered saline (PBS), followed by 30 ml of 4% wt/vol paraformaldehyde. They were postfixed for 24 h, cryoprotected in 30% sucrose and sectioned sagitally at 30 μm thickness on a freezing-sliding microtome. A one-in-six series of sections was stored in PBS with 0.02% sodium azide at 4°C until staining.

### Assessment of bleeds

Two methods were used to assess cerebral microbleeds, staining adjacent sections with hematoxylin & eosin (H&E) and Perl's Prussian blue. H&E staining was performed according to a standard procedure using Meyer's Hematoxylin and was also used to detect any other damage to the tissue. Prussian blue staining was performed using freshly prepared 5% potassium hexacyanoferratetrihydrate and 5% hydrochloric acid for 10 min. Sections were rinsed in water and counterstained with nuclear fast red, dehydrated, and mounted with Depex. Cerebral microbleeds were identified at a 20× magnification as cherry-red colored erythrocytes using H&E staining, and as blue deposits using Prussian blue staining. For each of these stainings, four mid-sagittal sections per mouse were analyzed blinded, with no identification of treatment group until after the data were obtained.

### Assessment of amyloid plaque load

For staining plaques with methoxy-X04 (Burgold et al., 2011; Burgess et al., 2014), sections were washed with PBS, incubated with 10 μM methoxy-XO4 (Tocris Bioscience) in 50% DMSO/50% NaCl 0.9%, pH 12 for 10 min and washed twice with PBS. A Metafer fluorescent VSlide Scanner by MetaSystems using a Zeiss Axio Imager Z2 acquired images with a 20x objective. The analysis was performed with ImageJ (National Institutes of Health). Four mid-sagittal sections from each mouse brain were analyzed blinded, with no identification of treatment group until after measurements were obtained. Data were obtained for total plaque number, individual plaque sizes, forebrain plaque-positive area fraction, and mean gray value per section as well as the mean gray value per thresholded plaque area were calculated. Automatic thresholding of plaques was performed by the following procedure: The average fluorescence in the cerebral peduncle of the cerebellum was defined as non-specific fluorescence, due to lack of plaques in this brain region, and subtracted from the image. The MaxEntropy function was used to identify the plaques and convert the image to a binary image. Watershed and fill hole functions were applied. From this the number of plaques, plaque area, plaque size, and mean gray values were calculated using the “analyze particles” plugin. A minimum size of 10 μm in diameter and 0.1 circularity was used to ensure specificity of plaque detection. The Aβ area fraction was determined by dividing the total plaque area by the area of the forebrain. CAA length was determined manually by a blinded observer, using ImageJ.

### Microglial assessment

Microglia were immunostained with anti-Iba1 antibody (Wako, JP 1:1,000) followed by incubation with an anti-rabbit secondary antibody AlexaFluor 568 conjugate (Invitrogen). Sections were then co-stained with 10 μM methoxy-XO4 (Tocris Bioscience) in PBS with 20% Ethanol before coverslipping. Images were obtained with a spinning disk confocal microscope (Nikon Diskovery) with a 20x objective acquiring z-stacks through the entire depth of the section. An experimenter blinded to experimental groups then manually outlined plaque areas and manually counted the number of microglia cell bodies within 20 μm of the perimeter of a plaque using Nikon NIS software. The total number of plaques analyzed was 179 total, 82 for the sham condition and 97 for the SUS condition. Three-four randomly chosen fields of view were captured from 2 to 4 sections from each mouse, resulting in a total number of between 14 and 23 plaques analyzed from each mouse.

### Statistics

Statistical analyses were conducted with Prism 7 software (GraphPad Software, USA). Values are always reported as mean ± SEM. Two-tailed *t*-tests with Welch's correction were used for all analyses except for the microglia analysis where a Deming multiple linear regression was performed.

## Results

### Study design to address the effects of SUS in aged APP23 mice

Using a previously established protocol (Leinenga and Götz, 2015), 10 21–22 month-old male APP23 mice were used. At this advanced age, APP23 mice have a massive plaque burden and also extensive CAA (Calhoun et al., 1999). Over a period of 8 weeks, 5 mice received 4 SUS treatment, and 5 mice received 4 sham treatments (with “sham” meaning an injection of microbubbles and anesthesia, but without applying ultrasound) (Figure 1B). After the last treatment, we waited for 4 weeks and then sacrificed the mice by perfusion fixation and analyzed them histologically. Mice were 23–24 months old at sacrifice.

### Safety of sonication in aged APP23 mice and effects on cerebral amyloid angiopathy (CAA)

**Figure 2:**
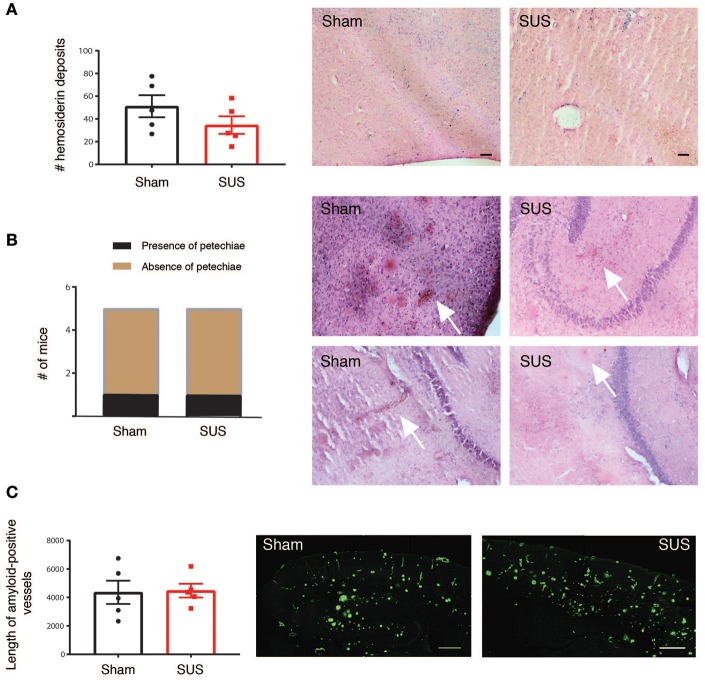
SUS in aged APP23 mice does not augment microbleeds and cerebral amyloid angiopathy (CAA). **(A)** Perl's Prussian blue staining detecting hemosiderin deposits reveals significant iron deposition in brains of aged APP23 mice, with no difference in numbers between sham- and SUS-treated mice. Blue spots label iron deposits from previous microbleeds. **(B)** Hematoxylin and eosin (H&E) staining reveals small acute cerebral microbleeds known as petechiae only in one sham- and one SUS-treated mouse in the sections analyzed. **(C)** Prominent amyloid-laden vessels (pathology known as CAA) are found in aged APP23 mice, with their length not differing between sham- and SUS-treated mice as determined with methoxy-XO4. Scale bars: 100 μm, data shown as mean ± SEM. White arrows indicate microbleeds.

Cerebral microbleeds are increasingly being recognized as a critical factor in the cognitive impairment in AD (Cacciottolo et al., 2016). In addition, anti-Aβ vaccines that are currently in clinical trials may pose the risk of microbleeds, so-called ARIA-H on magnetic resonance imaging (Sevigny et al., 2016). Therefore, we carried out a histopathological study to determine whether SUS causes microbleeds or hemorrhage in the APP23 mouse model with significant cerebrovascular amyloid and occurrence of cerebral microbleeds (Reuter et al., 2016). However, upon dissection, all brains were unremarkable without the appearance of surface bleeds. To detect microbleeds, sagittal sections were stained with H&E that detects newly formed bleeds with intact erythrocytes and with Prussian blue that can detect leftover brain iron from previously occurring bleeds (old lesions). While there were Prussian blue-reactive iron deposits in aged APP23 mice, there was no difference between treatment groups (Figure 2A). H&E staining did again not reveal differences between the treatment groups, as microbleeds that are also known as petechiae (<50 μm diameter) were found in one of the five sham-treated mice and in one of the five SUS-treated mouse for the sections analyzed. Erythrocyte clusters were detected by H&E staining that were around 100 μm in diameter and were confined to the cortex and thalamus in one sham-treated APP23 mouse and in the hippocampus of one SUS-treated APP23 mouse (Figure 2B). The risk of microbleeds is related to CAA, and therefore, the length of CAA was quantified on sections manually by a blinded observer. Again, no difference was found between the SUS- and sham-treated groups (Figure 2C).

### SUS treatment of 2 year-old APP23 mice does not reduce the total plaque area, but reduces the fraction of larger plaques

**Figure 3:**
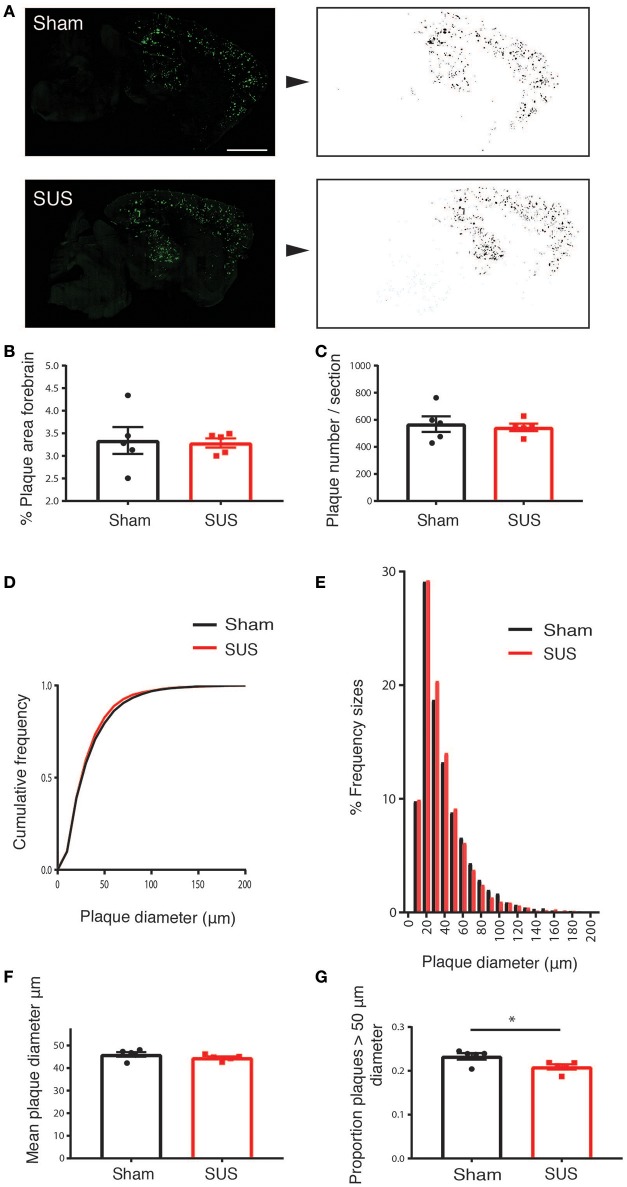
SUS treatment of aged APP23 mice does not reduce total plaque numbers, but the fraction of larger plaques. Methoxy-XO4 staining was used to visualize plaques (**A**, left). An automated threshold was applied and a binary image of plaques produced to obtain the size and number of plaques (**A**, right). This reveals no difference in the total amyloid-positive area **(B)**, and the number of plaques per section **(C)**. SUS shifts the cumulative frequency of plaque sizes toward smaller plaques **(D,E)**, without reducing the mean plaque size **(F)**. The proportion of large plaques is reduced, with large plaques being defined as having a diameter larger than 50 μm (accounting for about the largest 30% of plaques) **(G)**. Scale bar: 500 μm, mean ± SEM. *p < 0.05.

Methoxy-X04 is a derivative of Congo red and Chrysamine-G that contains no acid groups and is therefore smaller and much more lipophilic than Congo red or Chrysamine-G. Methoxy-X04 is fluorescent and stains amyloid plaques, tangles (that do not develop in APP23 mice), and CAA in postmortem sections of AD brain with good specificity (Klunk et al., 2002). We performed methoxy-XO4 staining for plaques in SUS- and sham-treated APP23 mice (Figure 3A). In these very old APP23 mice, after only four treatments, there was neither a reduction in the percentage plaque area in the forebrain nor the number of plaques per section (Figures 3B,C). However, we observed an effect on plaque size such that in the SUS-treated group, the cumulative frequency distribution of plaque sizes was shifted toward smaller plaques (Figures 3D,E), without affecting the mean plaque size (Figure 3F). When comparing the proportion of plaques that were larger than 50 μm^2^ (representing about the largest third of plaques), SUS reduced the proportion of these large plaques (*p* < 0.05; Figure 3G).

### SUS treatment reduces fibrillar amyloid

**Figure 4:**
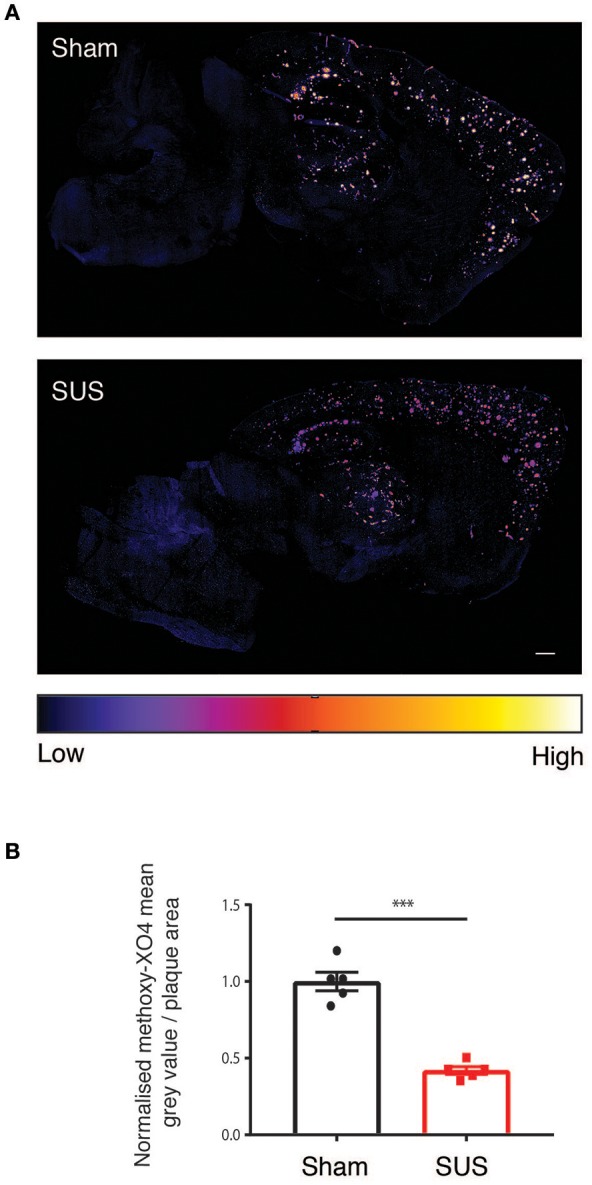
SUS treatment reduces fibrillar amyloid. **(A)** Sections treated with methoxy-XO4 render plaques fluorescent, followed by colorizing based on intensity reveals a reduction in fluorescence intensity in SUS-treated sections. **(B)** Scale bar: 500 μm, mean ± SEM. ***p < 0.001.

We next quantified the fluorescent staining of the amyloid plaques in the SUS- compared to the sham-treated mice because visual inspection revealed an obvious reduction in methoxy-XO4 fluorescence intensity on the sections from SUS-treated mice (Figure 4A). When a color is assigned to the mean gray value based on intensity, there was a visible 58% reduction in the SUS-treated group (*p* < 0.001) (Figure 4B).

Together, this indicates a reduction in amyloid in APP23 mice even at a very high age and after only four SUS sessions, and also, that SUS treatment does not elicit damage as shown by the absence of increased microbleeds and CAA pathology, underscoring the potential safety of a therapeutic ultrasound treatment targeting the amyloid pathology.

### SUS treatment increases the number of plaque-associated microglia

**Figure 5:**
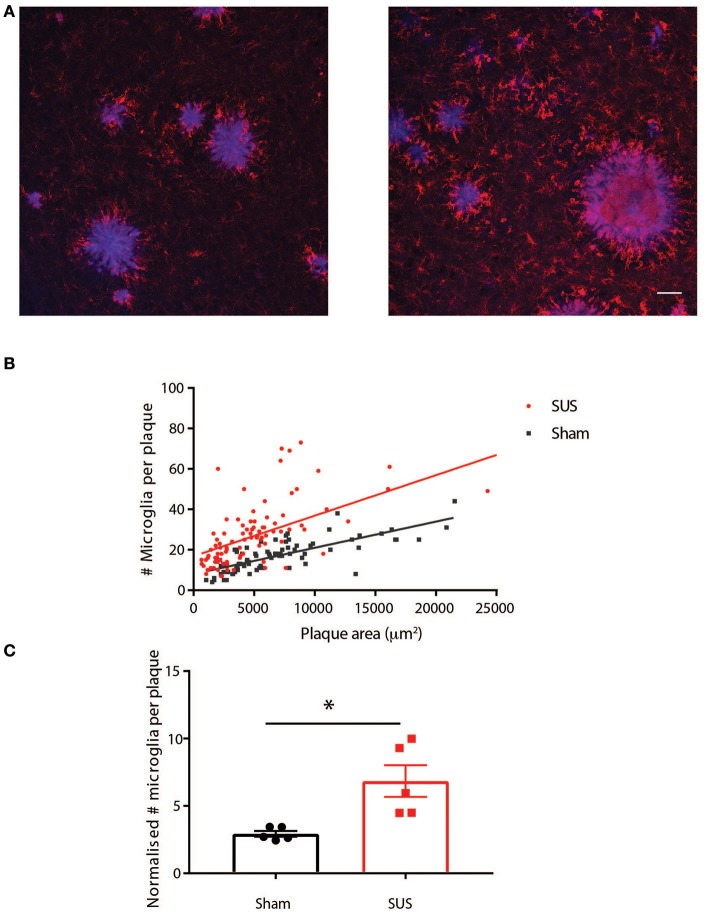
SUS treatment increases the number of plaque-associated microglia. **(A)** Co-labeling plaques (blue) and microglia (red) reveals an increase in plaque-associated microglia in SUS-treated mice. **(B)** A scatter plot of the data with Deming best fit regression lines reveals significant correlation between plaque size and number of microglia in plaque proximity, which is more pronounced for plaques in the SUS-treated mice. **(C)** Normalizing microglia number to plaque area and averaging per mouse reveals that more microglia per plaque in the SUS-treated mice. Scale bar: 50 μm, mean ± SEM. *p < 0.05.

To investigate possible mechanistic explanations for the reduced amyloid in SUS-treated mice, we determined the number of amyloid-associated microglia in the two groups as we have previously shown that SUS leads to microglia activation and increased phagocytosis of amyloid (Leinenga and Götz, 2015). To do this we performed co-staining for plaques and microglia with methoxy-XO4 and an anti-Iba1 antibody, respectively. This revealed an increased association of microglia with plaques in SUS-treated mice (Figure 5A). Plaque-associated microglia surrounding a total of 187 plaques of various sizes were analyzed. The number of plaque-associated microglia per plaque and plaque area was plotted as a scatter plot (Figure 5B). Plotting best-fit Deming regression lines for each group, it was found that there was a significant correlation between plaque area and the number of microglia in close proximity to plaques for both sham [*F*_(1, 80)_ = 113.9, *p* < 0.0001] and SUS [*F*_(1, 95)_ = 92.94, *p* < 0.0001] groups. The overall slopes were significantly different, indicating an interaction effect of the experimental group [*F*_(1, 175)_ = 6.227, *p* = 0.014]. These results show that plaques in the SUS-treated group have more plaque-associated microglia, and that in SUS-treated mice the the number of microglia per plaque is substantially increased for larger plaques. This conclusion was also supported by an analysis where normalizing for plaque size (microglia /1000 μm^2^ plaque) and averaging all plaques into one value per mouse revealed a significant increase in microglia per plaque in the SUS group compared to sham (Unpaired *t*-test with Welch's correction, *p* = 0.028; Figure 5C).

## Discussion

Therapeutic ultrasound is a novel potential treatment modality for human neurodegenerative diseases that may only employ an ultrasound sonication facilitated by biologically inert microbubbles or may be used in combination with a therapeutic agent (Leinenga et al., 2016). Several studies in mice have shown that the interaction of ultrasound with microbubbles transiently opens the BBB, allowing unidentified blood-borne factors and exogenously added antibodies or antibody fragments to enter the brain where they ameliorate the two defining pathologies of AD, Aβ and tau deposition, by an incompletely understood mechanism that involves microglia (Jordão et al., 2010, 2013; Leinenga and Götz, 2015). These studies have been done in mice that in the case of tau were quite young, and in the case of Aβ middle-aged, yet old enough to display an amyloid pathology, but not old enough to develop microbleeds and CAA. Here, we addressed the question whether SUS treatments in 2 year-old APP23 mice that are characterized by CAA and occasional microbleeds would augment these two pathologies. To detect microbleeds we used two complementary methods, one for acute bleeds (H&E staining) and the other for visualizing older, pre-existing lesions by detecting left-over hemosiderin from bleeds (Prussian blue). Interestingly, Prussian blue staining revealed iron deposits in all mice analyzed, irrespective of whether they were sham- or SUS-treated (Marinescu et al., 2017). Fresher lesions as determined by H&E staining were only found in one mouse each. Together this reveals that SUS treatment with the ultrasound parameters established used by us did not augment this pre-existing pathology. We had also analyzed CAA, finding neither an increase in vascular amyloid nor its clearance. This was not surprising as we had shown in APP23 mice that clearance of Aβ is mediated by microglia which are brain-resident and CAA mainly affects arterioles which are ensheathed in smooth muscle tissue (Calhoun et al., 1999) to which microglia would have limited access.

We further assessed the potential effect of SUS on amyloid load in the aged APP23 mice. As we had taken total brains for histological analysis, our study precluded a biochemical analysis. By histology, we made the intriguing observation that the cumulative frequency distribution of plaque sizes was shifted toward smaller plaques, but because the percentage plaque area was not reduced this may imply that upon SUS treatment the larger plaques are broken down into smaller plaques as the microglia perform their role of taking up Aβ. We performed stainings for microglia and plaques and found an increased interaction of microglia with plaques in SUS-treated mice, which is especially pronounced for large plaques which combined with our finding of reductions in larger plaques in SUS-treated mice suggests that microglia encircle and degrade large plaques in aged APP23 mice. These data suggests that activated microglia englobe and degrade large plaques based on the increased number of plaque-associated microglia in the SUS-treated group and the significant interaction effect when performing multiple linear regression of microglia number and plaque size which shows a more dramatic increase in the number of microglia for larger plaque sizes in the SUS-treated group compared to the sham condition.

When we visually inspected the methoxy-XO4-labeled plaques, this suggested a significant reduction in the fluorescent signal. Indeed, when a thresholding was performed this indicated a 58% reduction in the signal intensity indicative of a corresponding reduction of fibrillar Aβ in the SUS-treated mice.

In this study we compared SUS-treated mice which have openings of their BBB performed through the application of ultrasound concomitant with microbubble injection, and sham-treated mice which receive microbubbles but ultrasound is not turned on. Sham treated mice do not have openings of their BBB, and we believe are most similar to a placebo in clinical trial. It would have been interesting to test the effect of ultrasound without an injection of microbubbles; however, we aimed to model the treatment groups based on our earlier study that had used younger APP23 mice (Leinenga and Götz, 2015). It should be considered that previously, we had used a protocol with either 5 or 8 weekly treatments, followed by sacrifice (and analysis) either 3 or 1 day after the last sonication, respectively (Leinenga and Götz, 2015), whereas in the current study, we had spaced out the sonications much more, with four treatments over the course of 8 weeks, followed by sacrifice 4 weeks later. Considering this delay and the lower number of treatments in the context of a much more pronounced amyloid pathology our findings suggest that SUS causes reductions in amyloid pathology, even at an advanced stage. The number of plaque-associated microglia was higher in SUS-treated mice even 4 weeks following the last SUS treatment and even in these very old mice. It would be interesting to determine how Aβ that has dissociated from plaques might be cleared from the brain into the periphery. Of note, in a previous study in middle-aged APP23 mice we did not detect increases in plasma concentrations of Aβ immediately following SUS, which we believe to be due to microglia internalizing Aβ and degrading it (Leinenga and Götz, 2015).

It would have been interesting to determine if the reductions in pathology seen were sufficient to lead to increased performance of the mice in behavioral tests, or if too much damage has accumulated in 24 month old mice to see improvements following treatment. We did not perform behavioral tests because APP23 mice have an increased mortality (Ittner et al., 2010) making it impossible to obtain enough 24 month-old APP23 mice for sufficiently powered testing. More importantly, already wild-type mice at this age are significantly impaired in memory tests and we are currently addressing the effect of SUS in aged wild-type mice with memory impairment.

In regards to a possible application of the SUS technique in a clinical setting, one would envisage a treatment at an early MCI (mild cognitive impairment) stage when an amyloid pathology can be detected by either a PET (positron electron tomography) scan or a blood- or cerebrospinal fluid-based biomarker and before a point of no return has been reached. Therapeutic ultrasound targeting AD has already entered clinical safety trials (NCT02986932 and NCT03119961) and it remains to be seen whether this technology will stand up to its promises.

## Author contributions

GL and JG: Designed and monitored the experiments; GL: Performed the experiments and data analysis; JG and GL: wrote the manuscript.

### Conflict of interest statement

The authors declare that the research was conducted in the absence of any commercial or financial relationships that could be construed as a potential conflict of interest.
